# Discursive angles on the relationship in psychotherapy

**DOI:** 10.3389/fpsyg.2023.1198039

**Published:** 2023-06-19

**Authors:** Peter Muntigl, Claudio Scarvaglieri

**Affiliations:** ^1^Department of Translation, Interpreting and Communication, Ghent University, Ghent, Belgium; ^2^Section of German Language and Literature, University of Lausanne, Lausanne, Switzerland

**Keywords:** therapeutic relationship, conversation analysis, discourse analysis, the alliance in therapy, affiliation, alignment, empathy, ruptures

## Abstract

Research on the psychotherapy relationship has been dominated by quantitative-statistical paradigms that focus on relationship elements and their (evidence-based) effectiveness regarding the psychotherapy process. In this mini review, we complement this existing line of research with a discursive-interactional view that focuses on how the relationship is *accomplished* between therapists and clients. Our review highlights some of the main studies that use micro-analytic, interactional methods to explore relationship construction of the following elements: Affiliation, cooperation (Alignment), empathy and Disaffiliation-Repair. We not only provide a summary of important discursive work that provides a unique lens on *how* the relationship may be established and maintained, but also suggest that this kind of micro-analytic approach can offer more nuanced conceptualizations of the relationship by showing how different elements work together in a synergistic manner.

## 1. Introduction

There is overwhelming agreement that the therapeutic relationship is one of the essential ingredients for making therapy effective and promoting a healing context between the client and therapist. Over the past decades, vast amounts of research have offered support for this claim (Norcross, 2002; Norcross, 2011; Wiseman and Tishby, 2015; Norcross and Lambert, 2018). In psychotherapy research, the relationship is mostly characterized in affectual terms, as “the feelings and attitudes that counseling participants have toward one another” (Gelso and Carter, 1985: 159), and is seen as composed of a variety of elements such as empathy, collaboration, the alliance, rupture repair and others (see Norcross and Lambert, 2018). Further, relationship elements are often assessed in terms of subjective measures, behavioral observations or feedback questionnaires. *Interaction*-focussed research, on the other hand, views the relationship as a discursive accomplishment, constantly negotiated between participants, turn-by-turn (Pomerantz and Mandelbaum, 2005). Whereas psychotherapy research tends to be directed at *what* works (regarding *evidence*-based measures of effectiveness), interaction research is interested instead on *how*, for instance, a certain intervention is discursively performed in a given conversational context (Strong and Smoliak, 2018).

Psychotherapy researchers have argued that close interactional analysis can promise “to fill the gaps in psychotherapy theory by conceptualizing and describing the moment-by-moment exchange between therapist and client” (Stiles, 2008: 1). In this mini review, we offer a discussion of “how” discursive research may be able to fulfill this promise, by summarizing past studies on relationship construction and the discursive ways in which relationships are established and managed (e.g., as ‘close’ vs. ‘distant’). Rather than competing with psychotherapy research aims, interactional studies, should be viewed as complementary (Stiles, 2008). Thus, a discursive lens may “elaborate psychotherapeutic abstractions” such as the relationship (or a given aspect of theory) and, as a result, may also have the potential to demonstrate how and why different aspects of therapy and the therapeutic relationship contribute to helpful therapy. Qualitative, discursive approaches may also allow for insights into how different aspects of the therapeutic relationship combine and work together [e.g., alliance and collaboration/self-disclosure and emotional expression (Norcross and Lambert, 2018)], in contrast to quantitative research approaches that often treat them as separate, stand-alone practices (Norcross and Lambert, 2018, 311), although in real-time interaction neither therapist nor patient experiences or produces them separately.

## 2. The relationship in psychotherapy research

The therapeutic relationship is generally considered an – if not *the most* –important factor for successful therapy and much research has focused on its conceptualization and description. Drawing from psychodynamic research paradigms, Gelso and Carter (1985) have characterized the relationship in therapy as comprising three different components. First, therapy largely consists of actions that are geared towards getting therapeutic work accomplished, which includes setting goals and agreeing on tasks. This, according to Gelso and Carter, is the *working alliance* component of the relationship. Second, it is argued that aspects of the therapist-client relationship may largely involve projections “based on his or her own wishes and fears stemming from unresolved issues in the past” (Gelso, 2009, p. 255), known as a *transference-countertransference configuration*. Third, the *real relationship*, is defined “as the personal relationship existing between two or more people as reflected in the degree to which each is genuine with the other and perceives and experiences the other in ways that benefit the other” (Gelso, 2009: 254–55). A major challenge to this relationship model, as Gelso (2009) himself acknowledges, is that it generally does not find much support in postmodern circles, as it invites critique in terms of defining ‘reality’, who may act as arbiters of ‘reality’ and also whether ‘what is real’ can actually be known. Putting questions of reality aside, however, we find that the model is important due to its emphasis on the ‘task-based’ component of the therapeutic relationship. As Kozart (2002, p. 220) argues, “the clinical relationship is not merely a means to define clinical goals and implementing tasks; rather, the goals and tasks are the means to strengthen a relationship that has an intrinsically therapeutic effect.”; that is, in Kozart (2002) *ethnomethodological* view, relationships in the therapy setting are not so much accomplished as an explicit topic in interaction, but rather through clients’ and therapists’ joint, ‘common sense’ attention on working towards the achievement of therapeutic goals.

Alongside – and in certain respects diverging from – Gelso’s tripartite relationship model, quantitative-statistical paradigms have developed concepts and categories to differentiate aspects of the relationship and to assess them quantitatively in terms of being *demonstrably* or *probably* effective. Some of these elements include empathy, collaboration, the alliance and dealing with alliance ruptures (for a full list, see Norcross and Lambert, 2018). Whereas those approaches have been able to demonstrate that these aspects contribute significantly to good therapy outcomes, they have not shown *how* these elements are instantiated or even relate to each other (Horvath, 2006). To understand the process and the inner workings of relationship construction, we refer to studies that investigate interaction in therapy. Proceeding in this manner allows us to connect two approaches that have so far in general been treated as separate, one as stemming from a *quantitative*, the other from a *qualitative*-interactional paradigm.

For the remainder of this review, we provide a summary of the “discursive turn” in psychotherapy relationship research. For reasons of space, we restrict ourselves to studies on individual therapy (for interactional studies on the relationship in couple or family therapy see Muntigl and Horvath, 2016; Kykyri et al., 2019; Nyman-Salonen et al., 2021; for interpreter-mediated therapy Scarvaglieri and Muntigl, 2022).

## 3. The discursive turn in relationship research

It has long been recognized by linguistic scholars that language has a social, relational component (Malinowski, 1923; Bühler, 1934; Jakobson, 1960). Brown and Gilman (1960) influential paper on *power* and *solidarity* showed how certain language selections (e.g., *tu* and *vous*) may constitute relationships between speakers along those dimensions. Drawing from Goffman (1967) work on *face*, Brown and Levinson (1987) built extensively on Brown and Gilman’s initial observations, illustrating how speakers’ linguistic selections, which comprise *facework*, orient to various relationship dimensions (power, social distance and imposition of the face-threatening action). Scholars of social interaction have argued that talk itself is organized along relational terms, for example, to promote social solidarity and avoid conflict (Goffman, 1967; Davidson, 1984; Heritage, 1984; Sacks, 1987). This kind of (pro-social) organization, according to Enfield (2006: 399–400) goes even further to suggest that the (pro-social) organization recurrently found in talk, indexes an *affiliation imperative* that “compels interlocutors to maintain a common degree of interpersonal affiliation (trust, commitment, intimacy), proper to the status of the relationship, and again mutually calibrated at each step of an interaction’s progression.”

The psychotherapy relationship has become a Central topic in discourse studies (see Scarvaglieri et al., 2022). In this section, we briefly review some of the burgeoning areas of discursive research by focussing on aspects of the relationship pertaining to what conversation analysts have termed *affiliation* and *alignment* (Stivers, 2008; Steensig, 2020). According to Steensig (2020), these concepts represent different types of cooperative responses, with affiliation referring to the affectual level and alignment to the structural, task/goal-oriented level – the counterparts to these concepts, *disaffiliation* and *disalignment*, generally index a certain quality of *non*-cooperativeness. These concepts may be seen as ‘loosely connected’ to the alliance, with affiliation related to ‘interpersonal bonds’ (but also to Gelso’s *real* relationship) and alignment to tasks/goals. Our discussion will also address two other areas import for relationship accomplishment: empathy and disaffiliation-repair (or rupture-repair) sequences.

### 3.1. Affiliation

The therapeutic relationship has been called the “infrastructure of therapy” (Peräkylä, 2019: 273) that facilitates therapeutic work. From an interactional perspective, a central element of a functioning therapeutic relationship consists of affiliative actions by therapist and client. Following Stivers (2008), Stivers et al. (2011), p. 20, and Muntigl et al. (2013), affiliative actions orient towards the prior utterance in an agreeing, pro-social way. Affiliation can be understood as trust, commitment and intimacy (Enfield, 2006) and is related to the emotional agreement and the bond (Lindstrom and Sorjonen, 2013) created in interaction. Affiliative actions are “maximally pro-social when they match the prior speaker’s evaluative stance” (Stivers et al., 2011, 21).

Interactional research on affiliation in psychotherapy has discussed different methods by which therapists and patients (re-)establish affiliation. Affiliative actions in general orient towards the other person, by expressing and displaying agreement, understanding, support and positive feelings. In therapy, this can take the form of therapist’s relating to client’s narratives (Muntigl et al., 2014; Muntigl, 2022; Pawelczyk and Faccio, 2022) and expressing agreement. Frequently they will also reformulate the client’s experience to demonstrate understanding (Muntigl et al., 2012; Scarvaglieri, 2013) or point out specific aspects in the client’s behavior, narrative or expression that show them to be attentive and listening closely (Muntigl et al., 2020). Therapists may also use specific techniques, like solution-oriented questions (Kabatnik et al., 2022) to demonstrate that they are perceptive towards the client’s problems and reflective concerning possible solutions. Another way of relating to the client more closely is by using role referrals that address the client in a more personal way and thereby affiliate with them (Muntigl, 2022).

Overall, interactional research on affiliation has demonstrated the emphasis that therapists and clients put on affiliating with each other – as becomes especially clear by the numerous ways they work to ‘repair’ any previous disaffiliate moves (see below, 3.4). Through their actions, the participants thus express themselves in ways shown by traditional outcome oriented research: that a functioning therapeutic relationship is (seen as, treated as) vital for a therapeutic process that leads to good results.

### 3.2. Alignment

In interactional psychotherapy research, alignment has often been discussed in relation to affiliation, as referring to the organizational and sequential aspect of interaction. Alignment characterizes the participants’ mutual willingness and intention to cooperate, to pursue a common goal, and to work together in the same cooperative process. Therefore, when asking whether therapists and clients are aligning, we are in essence asking whether they are participating in the same activity, whether they are orienting to the same ‘task at hand’. Different from affiliation (or empathy, see below), alignment is thus not related to emotional aspects of interaction, but to the structural, task-based organization of interaction.

Research has shown that therapists frequently disalign with patients to pursue interactional goals related to the purpose of therapy. They for instance refuse to answer patients’ questions and instead point out the patient’s right and responsibility to decide on the direction of the session (Scarvaglieri, 2020). In other cases, therapists will change the projected interactive path – and thereby disalign with the patient –to present interpretations (Vehviläinen, 2003; Peräkylä, 2005, 2011), formulations (Muntigl et al., 2013) or explanations of the patients’ experience. Disalignment can also come about through longer passages of silence, i.e., one of the participants refusing to accept the turn and thereby not partaking in the projected activity. In those cases, just continuing the conversation can be a way of realigning on a formal, organizational level of interaction (Scarvaglieri, 2020).

Research has also shown that disaligning carries risks of weakening or jeopardizing the therapeutic relationship. Therapists therefore use a variety of measures to weaken the impact of disaligning actions in a variety of ways: framing disaligning utterances as statements not about facts but about their imagination (Muntigl and Horvath, 2014: 331); using hedges or “epistemic downgraders” (Muntigl and Horvath, 2014: 332) to weaken the contents of their proposition (Vehviläinen, 2003; Weiste et al., 2016): expanding the topic to facilitate agreement: or formulate suggestions in the form of a question (Scarvaglieri, 2020). Patients on the other hand, will also do considerable interactional work when disaligning with therapists (Guxholli et al., 2022), thereby showing the importance they also put on a functioning relationship.

### 3.3. Empathy

Empathy is considered to be a key relational element (Norcross and Lambert, 2018). In interactional terms, empathy is a social accomplishment between speakers in which one person tells of their troubles and another speaker goes ‘on record’ to display an understanding of the trouble. Going *on record* means that the understanding is demonstrated in an explicit fashion that usually references an emotional/cognitive state (Hepburn and Potter, 2007). Consistent with person-centered tenets, understanding targets the client’s frame of reference, thus preserving the client’s expert status regarding own experience and personal knowledge. The most common social actions that do empathic work are *formulations* that summarize or provide an upshot of client experience (Antaki, 2008; Muntigl et al., 2014). Actions that interpret, counter or sympathize with client troubles are generally not viewed as empathic (Hepburn and Potter, 2007; Muntigl, 2023). Empathy is achieved as a sequence of moves (Frankel, 2009; Muntigl et al., 2014; Ford and Hepburn, 2021). The first two moves, troubles telling + empathic response, have already been briefly discussed. The 3rd move, client feedback, shows how clients have understood the therapist’s understanding, generally via some form of assessment or dis/confirmation (Muntigl, 2023). Empathic sequences, when they unfold in an affiliative manner, are important sites for doing relationship work because they can produce what has been termed *empathic moments* (Heritage, 2011). For these empathic moments to occur, two conditions should be met. First, the therapist affiliates with the client’s troubles telling stance by displaying understanding and, second, the client ratifies this understanding through further affiliative displays. There is a growing body of discursive work on empathic responses in psychotherapy, (Voutilainen, 2012; Muntigl et al., 2014; Weiste and Peräkylä, 2014; Voutilainen et al., 2018; Nissen Schriver et al., 2019, 2022).

### 3.4. Disaffiliation-repair-sequences

Repairing disaffiliation (commonly known as ‘rupture-repair’ in psychotherapy research) is also considered to be a key relationship element (Norcross and Lambert, 2018). Forms of tension, reluctance, resistance, conflict, lack of trust, etc. are of course in no way unusual in psychotherapy and, in fact, tension (‘alliance rupture’) is even argued to play a pivotal role in doing productive therapeutic work (Bordin, 1994; Safran and Muran, 1996). Social actions that oppose or disagree with other’s points of view or in some way withdraw or disengage from certain interactional constraints may be viewed as potentially damaging social relations. This is because of the various implications that may arise from resistance or opposition: Recipients (i.e., persons to whom the resistance, opposition, etc. is directed at) may no longer feel supported, liked or appreciated [e.g., Goffman (1967) concept of *face* or Brown and Levinson (1987) *positive face*], thus leading to increased social distance. Thus, repairing these problematic moments will generally be seen as having relationship benefits, as trust, emotional support, ‘closeness’ can be restored in the process.

There is a growing number of discursive studies examining the relationship repair process from an interactional lens. For example, some studies have examined sequences involving client disagreement and the various practices therapists use to regain affiliation (Muntigl et al., 2013; Cardoso et al., 2020; Guxholli et al., 2021). Further, studies on emotion-focused therapy showed how therapist re-affiliation practices operated multi-modally, through various vocal (mirroring repeats, joint completions, second formulations) and non-vocal resources (e.g., nodding; Muntigl et al., 2013). Other studies have examined initial reluctance or opposition to engage in an in-session task (i.e., chair work), the interactional strategies emotion-focused therapists would use to get clients to comply with the proposal (Muntigl et al., 2020) and statements that incorporate the patient’s perspective into the therapist’s argumentation (Scarvaglieri, 2013; Pawelczyk and Faccio, 2022).

## 4. Discussion

Our mini review has briefly outlined some important discursive, interactional studies that have focussed on how various relationship elements are realized, *in situ*, at the micro-level of conversation. This research is also beginning to shed light on how different elements are achieved within the same interventions. For example, some research has begun to explore connections (similarities and differences) between affiliation and alignment/cooperation (Muntigl and Horvath, 2014; Scarvaglieri, 2020) or affiliation and empathy (Muntigl, 2023). More work is needed to identify and explain how these elements are jointly realized, discursively, and how a certain relationship quality is achieved and maintained in the process.

Another area of interactional research that is still in its infancy pertains to the non-vocal level and its importance for negotiating relationships. For example, it has also been argued that nonverbal synchrony can be a marker of ‘well-being’ (Nyman-Salonen et al., 2021) – see also Streeck (2009) for related discussions on the topic of synchrony. A recent study by Peräkylä et al. (2023) has begun to address this gap by showing how non-vocal resources such as body position and gaze direction work to display engagement or disengagement, thus providing a poignant picture of the relationship quality between persons at a given moment in time. To conclude, discursive studies not only provide an important lens on the multi-faceted ways in which relationships are achieved, they also provide a complement to existing work in psychotherapy research, showing how relationship elements form an integral part of talk and work together in a synergistic fashion.

## Author contributions

All authors listed have made a substantial, direct, and intellectual contribution to the work, and approved it for publication.

## Conflict of interest

The authors declare that the research was conducted in the absence of any commercial or financial relationships that could be construed as a potential conflict of interest.

## Publisher’s note

All claims expressed in this article are solely those of the authors and do not necessarily represent those of their affiliated organizations, or those of the publisher, the editors and the reviewers. Any product that may be evaluated in this article, or claim that may be made by its manufacturer, is not guaranteed or endorsed by the publisher.
